# Microarchitected Compliant Scaffolds of Pyrolytic Carbon for 3D Muscle Cell Growth

**DOI:** 10.1002/adhm.202303485

**Published:** 2024-01-02

**Authors:** Mohammadreza Taale, Barbara Schamberger, Miguel A. Monclus, Christian Dolle, Fereydoon Taheri, Dario Mager, Yolita M. Eggeler, Jan G. Korvink, Jon M. Molina‐Aldareguia, Christine Selhuber‐Unkel, Andrés Díaz Lantada, Monsur Islam

**Affiliations:** ^1^ Institute for Molecular Systems Engineering and Advanced Materials (IMSEAM) Heidelberg University Im Neuenheimer Feld 225 69120 Heidelberg Germany; ^2^ IMDEA Materials Institute Eric Kandel, 2 Getafe 28906 Spain; ^3^ Microscopy of Nanoscale Structures and Mechanisms (MNM) Laboratory for Electron Microscopy (LEM) Karlsruhe Institute of Technology Engesserstr. 7 D‐76131 Karlsruhe Germany; ^4^ Institute of Microstructure Technology Karlsruhe Institute of Technology Hermann‐von‐Helmholtz‐Platz 1 76344 Eggenstein‐Leopoldshafen Germany; ^5^ Department of Mechanical Engineering Universidad Politécnica de Madrid José Gutierréz Abascal, 2 Madrid 28006 Spain

**Keywords:** 3D printing, architected material, cell culture scaffold, compliant structure, pyrolytic carbon, skeletal muscle cells

## Abstract

The integration of additive manufacturing technologies with the pyrolysis of polymeric precursors enables the design‐controlled fabrication of architected 3D pyrolytic carbon (PyC) structures with complex architectural details. Despite great promise, their use in cellular interaction remains unexplored. This study pioneers the utilization of microarchitected 3D PyC structures as biocompatible scaffolds for the colonization of muscle cells in a 3D environment. PyC scaffolds are fabricated using micro‐stereolithography, followed by pyrolysis. Furthermore, an innovative design strategy using revolute joints is employed to obtain novel, compliant structures of architected PyC. The pyrolysis process results in a pyrolysis temperature‐ and design‐geometry‐dependent shrinkage of up to 73%, enabling the geometrical features of microarchitected compatible with skeletal muscle cells. The stiffness of architected PyC varies with the pyrolysis temperature, with the highest value of 29.57 ± 0.78 GPa for 900 °C. The PyC scaffolds exhibit excellent biocompatibility and yield 3D cell colonization while culturing skeletal muscle C2C12 cells. They further induce good actin fiber alignment along the compliant PyC construction. However, no conclusive myogenic differentiation is observed here. Nevertheless, these results are highly promising for architected PyC scaffolds as multifunctional tissue implants and encourage more investigations in employing compliant architected PyC structures for high‐performance tissue engineering applications.

## Graphical abstract

3D microarchitected pyrolytic carbon structures are fabricated using microstereolithography of a precursor resin, followed by carbonization. Novel, compliant carbon structures are also demonstrated. These scaffolds exhibit excellent biocompatibility and 3D cell colonization while culturing C2C12 cells and promoting myotube formation. The findings showcase the potential of using compliant carbon structures for innovative tissue engineering applications.

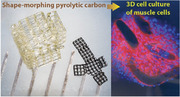

## 1 Introduction

Additive manufacturing, popularly known as 3D printing, has been gaining significant attention in the field of tissue engineering due to its ability to fabricate synthetic biocompatible scaffolds with intricate 3D and customizable geometries, which can effectively support cell growth and tissue formation.^[^ ^1^, ^2^, ^3^ ^]^ Various 3D printing methodologies, including material extrusion, material jetting, and vat photo‐polymerization, have been explored for scaffold fabrication. Additionally, the emerging field of bioprinting allows direct 3D printing of cells incorporated within a hydrogel ink while maintaining spatial configurations that mimic anatomical structures.^[^ ^4^ ^]^ These technologies construct complex 3D architectures with predefined interconnected pore geometries and sizes, typically through computer‐aided design and a layer‐by‐layer deposition approach. Despite rapid advancements, several challenges persist in additive manufacturing for scaffold fabrication. Achieving an optimal balance between part size, printing resolution, dimensional range, structural stability, and biocompatibility within a single fabrication mode remains elusive. For instance, the most commonly used syringe‐based methods (e.g., fused deposition modeling and 3D bioprinting) still lack fabrication precision and resolution compared to other additive manufacturing technologies. On the contrary, two‐photon polymerization offers the most precision and highest resolution in fabrication, which can potentially allow scaffold interaction at the cellular level.^[^ ^5^, ^6^ ^]^ However, it still lacks fabrication throughput, and the overall building volume is normally limited to less than 1 mm^3^. Although stereolithography and vat‐polymerization hold promise in terms of fabrication scale, precision, and resolution, their use of photo‐curable epoxy resins as printing materials often leads to sub‐optimal biocompatibility and cytotoxicity to cells.^[^ ^7^ ^]^

Carbon is a promising material for biomaterial scaffolds and biofabrication due to its biocompatibility, chemical inertness, and tunable mechanical and electrical properties.^[^ ^8^, ^9^, ^10^ ^]^ Among different carbon allotropes, carbon nanotubes (CNTs) and graphene have, in fact, gained popularity amongst different non‐polymeric tissue engineering scaffold materials, where culturing of skeletal muscle cells was also demonstrated.^[^ ^11^, ^12^ ^]^ However, due to the nanomaterial nature of CNTs and graphene, these materials can not be directly additively manufactured. The conventional approach for 3D structuring of carbon nanomaterials involves using a template‐based method, which involves growing or depositing carbon nanomaterials onto a 3D porous template, followed by the removal of the template material through etching.^[^ ^13^, ^14^, ^15^ ^]^ The drawback of this approach is that the shape of the 3D carbon material is constrained by the properties of the scaffold material. Alternatively, CNTs and graphene can be incorporated into a polymeric matrix to facilitate direct 3D printing.^[^ ^16^, ^17^ ^]^ However, a polymeric composite can compromise the inherent biological responses of pristine graphene or CNTs. Furthermore, the incorporation of graphene or CNTs has mostly been employed in extrusion‐based 3D printing, which, as mentioned earlier, lacks printing accuracy and resolution, often restricting 3D cell colonization. To achieve 3D printing of pure carbon with a structural resolution compatible with the desired tissue engineering application, the pyrolytic transformation of a structured polymeric precursor offers a viable solution. The feasibility of 3D‐architected pyrolytic carbon (PyC) has already been demonstrated at different length scales by combining different additive manufacturing processes with a subsequent pyrolysis process.^[^ ^18^ ^]^ For example, two‐photon polymerization leads to the fabrication of 3D‐architected PyC with a resolution down to hundreds of nanometers,^[^ ^19^, ^20^ ^]^ whereas stereolithographic 3D printing yields structural dimensions from sub‐100 µm to several millimeters.^[^ ^21^, ^22^ ^]^ However, the application of architected 3D PyC structures as 3D cell growth scaffolds remains majorly unexplored. A few studies have shown promising results in using architected 3D PyC structures in bone tissue engineering.^[^ ^23^, ^24^ ^]^ However, the pore sizes between lattice elements in these studies were significantly larger (>300 µm), restricting from achieving 3D cell colonization.

In this work, we introduce microarchitected 3D PyC structures as potential biomaterial scaffolds and study their interactions with skeletal muscle cells for 3D cell growth, which is, to the best of our knowledge, a first‐of‐its‐kind investigation in this field. Micro‐stereolithography‐based 3D printing and subsequent pyrolysis are used here for the reliable fabrication of microarchitected 3D PyC scaffold with geometrical feature sizes comparable to those of skeletal muscle cells, which typically vary from a few tens of microns to 100 µm. While stationary rigid scaffolds are commonly employed in tissue engineering applications, we also developed a novel concept for fabricating shape‐morphable microarchitected 3D PyC scaffolds. In this context, “shape‐morphing” refers to the capacity of structures to change their spatial arrangement through mechanical or manual manipulation. It should be noted that PyC is inherently brittle, constraining its natural shape‐morphing potential. To overcome this challenge, we employed a compliant mechanism design‐based route for enabling the shape‐morphing capabilities of microarchitected 3D PyC structures. To our knowledge, this is the first report on introducing compliant design principles to microarchitected 3D PyC structures. The dynamic nature of the compliant scaffolds is expected to enhance their potential to mimic the mechanical cues that the cells experience in the natural environment.

This work focuses on exploring the fabrication capabilities of both rigid and compliant microarchitected 3D PyC structures and characterizing the properties of the obtained PyC material. Additionally, we investigate the interaction of skeletal muscle cells with the fabricated microarchitected 3D PyC structures to evaluate their biocompatibility and ability for 3D cell growth based on their fabrication conditions. The findings of this study provide valuable insights into the potential of 3D‐printed PyC structures as advanced skeletal muscle scaffolds. Furthermore, the introduction of microarchitected compliant PyC structures opens up new opportunities for precisely architecting the microenvironment of muscle cells, encouraging further discussions on future research directions and the potential impact in this field.

## 2 Results and Discussion

Microarchitected compliant 3D PyC structures were designed and fabricated. To this end, research on increasing complexity needs to be performed. First, 3D architected PyC structures were fabricated using a top‐down micro‐stereolithography process, followed by carbonization in a nitrogen environment. A schematic of the micro‐stereolithography process is shown in Figure S1, Supporting Information. Details of the fabrication process are provided in the Experimental Section.

### 2.1 3D Architected Pyrolytic Carbon

**Figure 1:**
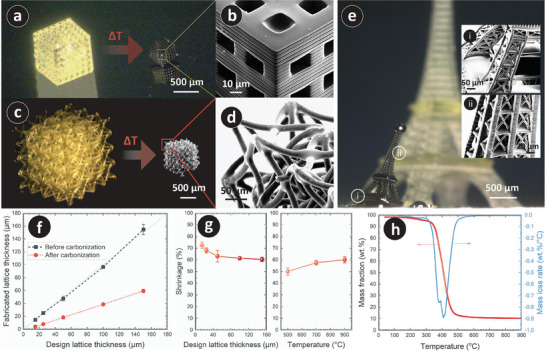
a) 3D printed architecture featuring cubic unit cells before and after carbonization. b) SEM of the architected PyC with cubic unit cells, showing the sequential print lines as evidence of layer‐by‐layer fabrication mode. c) An example of 3D printed auxetic metamaterial before and after pyrolysis, demonstrating the preservation of design complexity and structural integrity after pyrolysis. d) SEM image of the PyC auxetic metamaterial, illustrating the cohesive integrity of the PyC metamaterial, conforming to the original structure. e) Eiffel Tower before and after pyrolysis, as an example of the feasibility of fabricating a multi‐scale complex architecture. Insets present SEM images of marked spots (i) and (ii) on the PyC Eiffel Tower. f) Dimension of microlattices before and after carbonization. g) Dependency of shrinkage on design lattice thicknesses and pyrolysis temperature. For design lattice thickness, the pyrolysis temperature was kept constant at 900 °C. A design lattice thickness of 150 µm was used for studying the temperature‐dependent shrinkage. h) TGA and the first negative derivative of the TGA curve of the precursor material.

To understand the limits of the fabrication process, we fabricated structures with simple cubic unit cells, varying the lattice thickness from 5 to 150 µm, considering that the resolution of the stereolithographic printer was 2 µm. It was observed that architected structures with a lattice thickness below 15 µm could not be fabricated reliably and consistently. The merging of several lattice elements was observed due to overexposure, leading to undefined unit cells. An example of a fully defined architected structure with cubic unit cells before and after carbonization is shown in **Figure** **1**a, while Figure 1b displays a scanning electron microscopy (SEM) image of the 3D PyC, illustrating fully defined lattices with printing layers as the signature of the layer‐by‐layer fabrication process and pore structures.

As expected and previously reported for PyC structures,^[^ ^20^, ^23^, ^25^ ^]^ the pyrolysis process resulted in significant geometrical shrinkage due to the release of gaseous compounds during the thermochemical decomposition of the precursor resin. This shrinkage allowed for the PyC lattice thickness to be significantly smaller than the fabrication capabilities, as shown in Figure 1f. For example, a fabricated structure with a design lattice thickness of 15 µm resulted in a PyC lattice thickness of 4.1 ± 0.4 µm after pyrolysis. The degree of shrinkage depended on the lattice thickness of the precursor and the pyrolysis temperature, as plotted in Figure 1g. The release of the gaseous byproducts occurs through degassing from the surface.^[^ ^26^ ^]^ Therefore, a higher surface area leads to a higher degree of degassing. Smaller lattice thicknesses provided a higher surface area‐to‐volume ratio, leading to a higher degree of shrinkage, as observed in our study. For instance, for a final pyrolysis temperature of 900 °C, shrinkage varied from 73 ± 3% to 60 ± 2% for design lattice thicknesses varying from 15 to 150 µm. On the other hand, increasing the pyrolysis temperature from 500 to 900 °C resulted in an enhanced extent of shrinkage from 50 ± 3% to 60 ± 2% for the lattice thickness of 150 µm. The temperature‐dependent shrinkage was mainly attributed to the mass loss over the temperature. To assess the mass loss, we performed a thermogravimetric analysis (TGA) of the precursor resin. The TGA result (Figure 1h) showed that the major mass loss occurred within the temperature range of 350 to 480 °C, attributed to the thermochemical decomposition of the precursor and the subsequent release of volatile compounds.^[^ ^27^, ^28^ ^]^ The significant and rapid mass loss, as indicated by the first negative derivative of the TGA curve, explained the significant shrinkage observed at 500 °C. Further increasing the temperature yielded a gradual mass loss, as evident in the TGA curve, resulting in an additional 7% shrinkage for a pyrolysis temperature of 700 °C. Beyond 700 °C, the mass loss was minimal. At this temperature, carbon atoms were reported to condense and rearrange into short and mid‐range graphene layers,^[^ ^29^ ^]^ which could cause additional shrinkage at higher temperatures. An additional 3% shrinkage when increasing the pyrolysis temperature from 700°C to 900 °C could be attributed to such phenomena. Nevertheless, it is important to note that the dimensions of the architected PyC material achieved here are significantly smaller than reported values using stereolithography processes, as the reported smallest lattice thickness to date is ≈50 µm.^[^ ^18^ ^]^

To demonstrate the intricate design complexity achievable through our fabrication process, we also fabricated additional structures. Figure 1c showcases an auxetic metamaterial with a design lattice thickness of 50 µm before and after carbonization. The SEM image of the carbonized metamaterial (Figure 1d) depicts the conformal integrity and structural stability of this intricate geometry post‐carbonization. In order to exemplify the feasibility of fabricating multi‐scale constructions using PyC, we selected the iconic Eiffel Tower in Paris, France. The Eiffel Tower represents an ideal example of a multi‐scale structure, with its intricate lattice distribution at different dimensions contributing to its designation as a “wonder of the world.” We successfully fabricated a scaled model of the Eiffel Tower, which featured a total height of 6 mm and a minimum design thickness of 20 µm. Both resin and the PyC Eiffel Tower are shown in Figure 1e. Notably, the carbonized Eiffel Tower displayed a slight inclination, possibly due to its considerable height and relatively thin lattice elements. These factors might have caused distortions during the thermochemical decomposition as the materials go through a semi‐solid transition phase. Nevertheless, the results are highly promising, as the pyrolysis process successfully retained the overall geometry despite such a complex multiscale construction, featuring a minimum lattice thickness of ≈6 µm, even after encountering challenges resulting in the “inclined Eiffel” phenomenon. The structural integrity of this large multiscale construction could be observed in the SEM images, as presented in Figure 1e(i,ii). While our study exhibited successful and exciting fabrication results and offers valuable insights, further extensive research is required to comprehensively evaluate the carbonization behavior of multi‐scale architectures with varying levels of complexity. This investigation extends beyond the scope of our current study, emphasizing the need for future dedicated research efforts in this area.

### 2.2 Material Properties of Microarchitected Carbon

**Figure 2:**
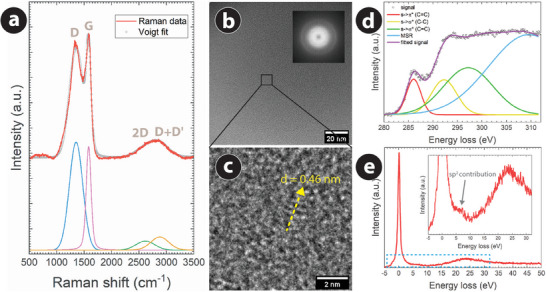
a) The Raman spectrum of architected PyC fabricated at the carbonization temperature of 900 °C. The Raman spectrum was deconvoluted into D, G, 2D, and D + D′ peaks using Voigt peak fitting for a better understanding of the carbon microstructure. b) TEM and c) HRTEM images of an architected PyC. The PyC sample was prepared using the FIB milling of an architected PyC. The inset of (b) shows the FFT of the TEM image. The HRTEM image further indicated the graphene layers with a *d*‐spacing of 0.46 nm. d) Electron energy loss spectrum (EELS) of architected PyC in the C‐K edge region and its deconvolution into different carbon bonds. e) Low loss EELS spectra of PyC, showing the magnified portion within −5 to 30 eV in the inset, indicating the presence of sp^2^ carbon.

The architected PyC featured a microstructural characteristic resembling glass‐like carbon, as devised from the Raman spectroscopy analysis. The Raman spectrum of the architected PyC material (**Figure** **2**a) obtained at 900 °C featured two prominent peaks centered around 1350 and 1585 cm^−1^, which are typical for carbon materials.^[^ ^30^, ^31^, ^32^ ^]^ The peak at around 1350 cm^−1^, assigned as the D‐band, corresponds to the A_1g_ mode, indicating an in‐plane breathing vibration associated with the disorder in the carbon matrix.^[^ ^33^ ^]^ The peak at around 1585 cm^−1^, denoted as the G‐band, corresponds to the Raman‐active *E*_2*g*_ in‐plane vibration mode, in other words, the active graphitic mode of the carbon. Therefore, the intensity ratio of the D‐band and the G‐peak (I_D_/I_G_) signifies the degree of graphitization within the carbon matrix. The intensity ratio of the architected PyC was measured as 0.9, suggesting the presence of a substantial number of graphitic planes within a highly amorphous carbon matrix in the architected PyC. The Raman spectrum was deconvoluted using Voigt fit to further evaluate the degree of disorder and graphitization. The Voigt fit resulted in a slight left‐shift of the G‐band (at 1580 cm^−1^), which could be because of the merging of the G‐band and D′‐band, originating from a high‐order of disorder.^[^ ^34^ ^]^ The crystallite size (L_a_) of the architected PyC, calculated from the Raman spectrum, was approximately 7.8 nm, similar to the turbostratic structure observed in glass‐like carbon materials.^[^ ^35^ ^]^ Furthermore, a broad peak around 2650 cm^−1^, assigned as the 2D‐peak, with a full width at half maximum of 407 cm^−1^, supports the highly amorphous nature of the PyC with a small graphitic content.^[^ ^34^ ^]^

The highly amorphous nature of the architected PyC was further confirmed by the transmission electron microscopy (TEM) study. Cross‐sectional TEM analysis revealed a predominant distribution of amorphous carbon with a small amount of loosely ordered layered material having a measurable amount of sp^2^ character (Figure 2b). The corresponding Fast Fourier Transform (FFT), as shown in the inset of Figure 2b, also signified the mostly amorphous character of the material, as no dedicated reflections were contained in the FFT, and only a faint ring was observable. Upon closer inspection using high‐resolution TEM, small areas with distinguishable lamellar contrasts were observed, as shown in Figure 2c. The distance between these ordered contrast features was measured to be 0.46 nm, estimated from a linescan along the ordered features (Figure S3, Supporting Information), resembling an enlarged inter‐layer distance of graphitic carbon. The PyC material often features microstructural defects and material impurities,^[^ ^29^ ^]^ which might be the case for our PyC material as well. These imperfections might have contributed to the enlargement of the interplane distances, as it could be often observed in graphite intercalation compounds^[^ ^36^ ^]^ or stacked multilayer graphene oxide.^[^ ^37^ ^]^

Further clarification of the partly graphitic nature of the PyC material was achieved through electron energy loss spectroscopic (EELS), which provided additional evidence consistent with the Raman spectroscopy. While a detailed quantification of the amount of sp^2^‐hybridized carbon structures is beyond the scope of this paper, the fingerprints recorded around the C‐K edge (Figure 2d) and the low loss energy (Figure 2e) are clear indications for sp^2^‐hybridized carbon, as visible from the deconvoluted signal at 285 eV (transition from s→ π* and indication for sp^2^ hybridized C = C bonds) and the shoulder at ≈5 eV in the low loss spectrum, as shown in the inset of Figure 2e. It should be mentioned here that the microstructural characteristics obtained from the Raman and TEM analysis are well consistent with other pyrolytic carbon materials obtained from the pyrolysis of micro/nano‐manufactured epoxy precursors.^[^ ^22^, ^23^, ^38^, ^39^ ^]^

### 2.3 Mechanical Properties of 3D Printed Pyrolytic Carbon

**Figure 3:**
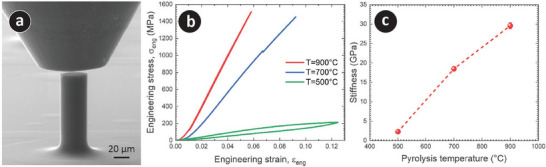
a) Microarchitected 3D PyC micropillar used for micromechanical testing. This particular example is for the sample obtained at the pyrolysis temperature of 900 °C. b) Representative stress–strain curve for PyC micropillars obtained at different pyrolysis temperatures (*T*). c) Stiffness of PyC materials at different pyrolysis temperatures.

We characterized the material stiffness of the PyC materials by performing compression tests on 3D PyC micropillars with a design diameter of 100 µm. The PyC micropillars are shown in **Figure** **3**a and Figure S4, Supporting Information. Representative engineering stress‐engineering strain curves for the micropillars are presented in Figure 3b. The initial shoulder in the stress–strain curves corresponds to the initial stages of deformation, which is common in micropillar compression until full contact is established between the top of the pillar and the flat punch. Once full contact was established, the micropillars processed at 900 °C exhibited a fully linear elastic behavior (the loading and unloading segments of the test fully overlap, as presented in Figure 3b; Figure S5, Supporting Information), with no signs of cracks or buckling up to the maximum applied load of ≈1.4 N (σ_eng_ ≈1.50 GPa). The loading and unloading on the PyC micropillar can be observed in Movie S1, Supporting Information. On the other hand, the micropillars carbonized at 700 °C showed some signs of cracking, such as the small pop‐in event (Movie S2 and Figure S4c, Supporting Information), after a fully linear elastic loading. These micropillars experienced a complete brittle fracture at a load of ≈1.3 N (σ_eng_ ≈1.45 GPa). These discontinuities and cracking events could be attributed to the existing surface defects on the PyC micropillars at 700 °C (see Figure S4b, Supporting Information). Finally, the micropillars processed at 500°C exhibited a non‐linear, fully elastic behavior (see Movie S3, Supporting Information). Moreover, the engineering stress‐strain curves displayed a noticeable hysteresis loop (see Figure 3b; Figure S5, Supporting Information), characteristic of a viscoelastic behavior like the one found for many polymeric materials. In this particular case, the pillars were deformed to a maximum strain, ε_eng_ ≈12.5%, corresponding to the stress of σ_eng_ ≈212 MPa, without any signs of cracking.

The compressive elastic moduli or stiffness of the micropillars were calculated from the slope of the linear part of the loading curves and plotted as a function of pyrolysis temperature in Figure 3c. The stiffness of the PyC materials increased from 2.29 ± 0.14 GPa for 500 °C pyrolysis temperature to 29.57 ± 0.78 °C for 900 °C. The low standard deviation in the measurement also signified the high reproducibility and uniformity of the fabricated samples. The mechanics of these PyC structures are beyond the scope of this paper and of consideration for our future publications. However, our initial assessment is that the temperature‐dependent variation in the mechanical behavior of the PyC materials can be attributed to the evolution of the material composition and microstructural arrangement of the PyC material with pyrolysis temperature. As shown in the TGA curve (Figure 1h), the thermal decomposition of the precursor resin was completed by 500 °C. At this temperature, it is highly probable that several heteroatoms from the precursor resins were still present in the carbon‐rich matrix,^[^ ^29^, ^40^ ^]^ which could be attributed to the polymer‐like hysteresis loading–unloading curve. However, the carbon atoms at this point were entirely disordered,^[^ ^29^, ^40^ ^]^ contributing to the high strength and elastic modulus. As the pyrolysis temperature increased, the heteroatoms were expected to gradually escape the carbon matrix, and the carbon atoms were expected to condense, rearrange, and start forming graphitic layers. Such transformations might have contributed to the enhanced modulus and stress‐strain behavior of the PyC materials at higher temperatures. The elastic behavior of the micro/nano‐architected PyC materials obtained at 900 °C was also reported by Zhang et al.^[^ ^38^ ^]^ However, in their case, the maximum diameter of the PyC micropillars was 12.7 µm and featured an aspect ratio of 1.4–1.8, and the behavior of the PyC micro/nanopillars was mainly attributed to the size effect. In comparison, the diameters of the PyC micropillars in our study were 30–40  µm, with an aspect ratio up to 3.0 (see the table in Figure S4, Supporting Information). The scaling effect might have also influenced the elastic behavior of our micropillars. However, the maximum strain here was ≈12.5%. Further experiments need to be done to understand the behavior in an extended strain range. Furthermore, the effect of aspect ratio also needs to be studied extensively. Nevertheless, it is worth mentioning here that the compressive strength and stiffness of the PyC micropillars were comparable or even superior to other high‐performance micro/nanostructures, including metal nanopillars,^[^ ^41^, ^42^ ^]^ epoxy nanocomposite,^[^ ^43^ ^]^ and nanolattice‐based metamaterials.^[^ ^44^ ^]^

### 2.4 Fabrication of Microarchitected Compliant Carbon Structures

Pyrolytic carbon is stiff and brittle in nature. It is extremely challenging, if not impossible, to change the geometrical shape of PyC material due to the inherent stiffness. To address this challenge, we employed a compliant mechanism design‐based approach. Geometrical structures with hinges were designed where the units were connected with revolute joints involving toroidal rings and shafts with controlled gaps, enabling compliant characteristics and thereby allowing the shape‐changing capability through these revolute joints. As each unit of the designed structures was not directly attached to another, it was essential to use support structures during the printing process. The support structures used in our experiments had a cylindrical shape with a conical head on the top. An example of a printed structure after printing is shown in Figure S6a, Supporting Information, whereas Figure S6b, Supporting Information, shows the distribution of the support structures under a structural unit. It is important to note that the support structures must be removed before the pyrolysis step, as carbonization with the supports in place can lead to structural deformations and failures due to non‐symmetric tensile forces exerted by the supports during pyrolysis‐induced shrinkage (Figure S6c,d, Supporting Information). To facilitate easier removal of the support structures, the size of the contact point of the supports with the designed structure, that is, the tip of the conical head, should be minimal. In our microstereolithography setup, a contact point of 30 µm was found to yield reliable and repeatable fabrication of the compliant structures. Furthermore, the height of the support structures also played a role here. Taller supports tended to yield easier removal, but they also translate to the increased aspect ratio, which tends to buckle after a critical value (which was not characterized in this study), affecting the printability of the desired resin structures. Therefore, there needed to be a trade‐off between printability and support removability when determining the support heights. In our experimental setup, for our designed compliant structures, the optimized support structures featured a height within the range of 200 to 400µm and a diameter of 50–60 µm. The conical head had a length of 60 µm. Upon fabrication, the support structures featuring these optimized dimensions could be removed manually with a sweep of a Q‐tip cotton swab.

**Figure 4:**
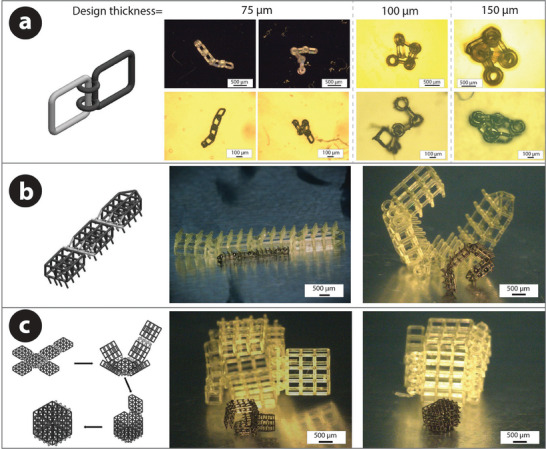
Examples of 3D printed compliant structures achieved through the designs of revolute joints. The left column displays the CAD designs of the respective compliant structure. a) Chain structures with different design thicknesses. The top columns present the compliant resin structures, and the bottom shows the compliant carbon structures. b) Caterpillar. c) Origami box structure. Different configurations of these structures are illustrated here for both resin and PyC structures to demonstrate the capability of achieving different shapes from the fabricated PyC structures.

The support dimensions also have an impact on the minimal feature size of the compliant structures. As the tip of the conical head of the supports was 30 µm, the lattice element of the design structures must be larger than that. Given the optimized support structures, we were able to achieve reliable and repeatable fabrication of compliant structures with a minimum lattice element thickness of 50 µm, as shown in Figure S7, Supporting Information. However, it should be mentioned that support removal from compliant structures with a 50 µm lattice thickness was highly complicated and often resulted in the catastrophic destruction of the structures. Reliable and repeatable support removal to achieve free‐standing compliant structures was achieved for a minimum design lattice thickness of 75 µm, as shown in **Figure** **4**a. The support removal further facilitated free‐form shape‐morphing of the structures through their revolute joints. The compliant resin structures were carbonized to convert them to PyC, while retaining their shape‐morphing capabilities, albeit with a significant size reduction due to isotropic shrinkage. The minimum PyC lattice thickness achieved was approximately 30 µm, obtained from the pyrolysis of the resin structure with a design thickness of 75 µm. Figure 4 showcases three examples of compliant PyC structures: a chain (a), a caterpillar (b), and an origami box (c). The corresponding CAD designs and resin structures were also included in Figure 4. Different configurations of the structures were presented here to illustrate that the geometrical shape of the structures could be changed. It should be noted that these configurations were achieved through manual manipulations of the structures, leveraging their shape‐morphing capabilities through compliant designs; no self‐responsive shape‐morphing is illustrated here.

### 2.5 Muscle Cell Culture within PyC Scaffolds

**Figure 5:**
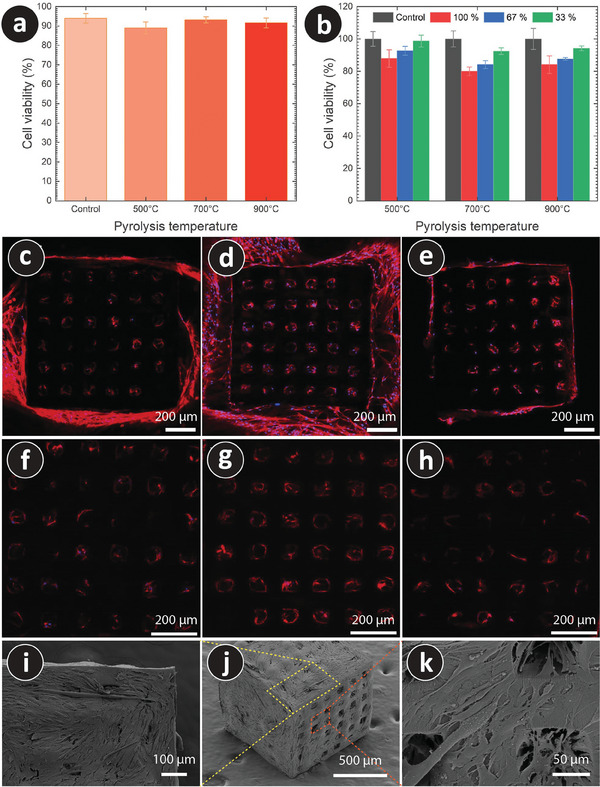
Biocompatibility of the architected 3D PyC scaffolds. a) The percentage of live cells out of the total number of cells after staining with calcein/PI. The C2C12 cells were cultured in the presence of carbon structures. b) Cytotoxicity assay on PyC structures at different temperatures using a metabolic activity of methylthiazolyldiphenyltetrazolium bromide (MTT). Different colors in the graph present the concentration of extract media. c–h) Fluorescence images of C2C12 cells on architected 3D PyC structures obtained at 500 °C (c,f), 700 °C (d,g), and 900 °C (e,h). The nuclei were stained with Hoechst (blue), and actin fibers were visualized with phalloidin (red). Fluorescence imaging was obtained in optical sections approximately 40 µm (c–e) and 43 µm (f–h) from the surface of the material. i–k) SEM images of the C2C12 cells on the architected 3D PyC structure (pyrolyzed at 500 °C) after 22 days of culturing.

We cultured C2C12 cells within 3D microarchitected PyC scaffolds to evaluate their biocompatibility and capability for 3D cell growth. C2C12 cells are often used as model cells in muscle research due to their mononucleated and spindle‐shaped myoblasts, which later differentiate into multinucleated myotubes, mimicking the process of muscle fiber formation in vivo.^[^ ^45^ ^]^ Following this common practice, we also used C2C12 cells in our study. The biocompatibility of the PyC scaffolds was examined using methylthiazolyldiphenyltetrazolium bromide (MTT) metabolic activity and live‐dead staining of cells in direct contact with the PyC materials. **Figure** **5**b shows the results of the PyC structures at 500, 700, and 900 °C using an MTT assay, demonstrating that all structures were biocompatible. A live‐dead assay was also performed on skeletal muscle cells in direct contact with the structures to investigate the biocompatibility of the PyC materials further. The ratio of live to dead cells were measured after calcein/PI staining and are plotted in Figure 5a for PyC scaffolds obtained at different pyrolysis temperatures. No significant sign of toxicity to muscle cells was seen on either the gloss or the structures.

Actin fiber significantly impacts intracellular motility, cell adhesion, and mechanical properties.^[^ ^46^ ^]^ Therefore, we studied the cellular actin arrangement of the C2C12 on the carbon lattices. The fluorescence images of phalloidin, as presented in Figure 5c–h, revealed dense bundles of filamentous actin in the cytoskeleton. The cells mainly covered the structures in the periphery. The actin fibers within the cultured cells appeared to be randomly distributed in the cytoskeleton. Interestingly, the pores were populated by skeletal muscle cells at least up to 43 µm from the surface of the material after seven days of culture, suggesting achieving 3D colonization, which is essential for many tissue engineering applications. 3D cell colonization was further confirmed by an SEM investigation. Figure 5i–k presents SEM images of the biohybrid construct after 22 days of cell culturing. The cells completely covered the PyC scaffold and exhibited an elongated morphology, indicative of the preferable proliferation of the cells on the PyC surface. Furthermore, cells were observed to grow within the pores and also briding on the pores, suggesting that the microarchitected 3D PyC supports 3D skeletal muscle cell growth. The pore dimension of the 3D PyC scaffold achieved here (≈55 µm in this case) was comparable to the size of C2C12 cells,^[^ ^47^ ^]^ which could have facilitated the 3D cell colonization. Furthermore, cells on pores exceeding 3.5 µm can generate robust actin bundles, serving to bridge and reinforce the contact zone of the cells.^[^ ^48^ ^]^ This can be seen in SEM images (Figure 5i–k), where the cell bridged the pore's opening.

**Figure 6:**
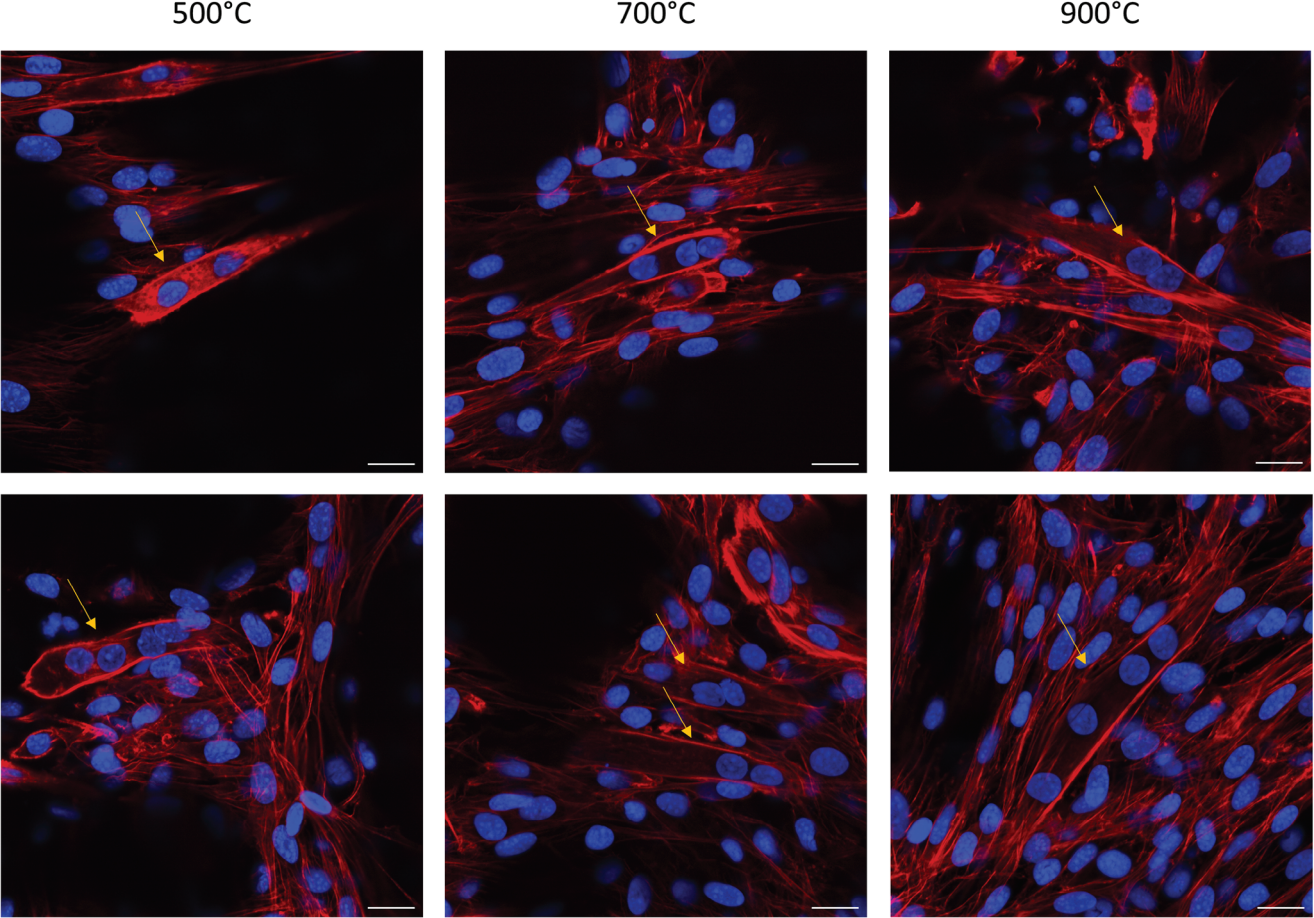
Fluorescence images show C2C12 cells on an architected 3D PyC structures pyrolyzed at 500, 700, and 900 °C. The nuclei were stained with Hoechst (blue), while actin fibers were stained with phalloidin (red). The images confirm the formation of multi‐nucleated cells, resulting from the fusion of mononucleated myoblasts with others. The yellow arrows highlight some of the multi‐nucleated myoblasts. The scale bars are 20 µm. High‐resolution imaging has been conducted solely on a single sample.

One of the key criteria of skeletal muscle cell scaffolds is their ability to induce the formation of myotubes, which are multinucleated fibril structures resulting from the fusion of parent myoblasts.^[^ ^49^ ^]^ Therefore, we investigated the potency of our architected PyC structures in inducing the formation of multi‐nucleated myoblasts by analyzing the fluorescence of stained nuclei and actin fibers with Hoechst and phalloidin, respectively. **Figure** **6** presents the results of very few myotube formations on PyC scaffolds prepared at different pyrolysis temperatures. Furthermore, we performed a fluorescent investigation for the formed myosin using anti‐myosin/Alexa Fluor 488 on the 3D PyC scaffolds (Figure S9, Supporting Information). These results show that myotubes were formed on all the 3D PyC scaffolds obtained at different temperatures, and they were well‐developed and randomly oriented on the surface of the scaffold. Nevertheless, there was a notable absence of any particular orientation among the limited myotubes formed on our structures. It has been demonstrated that substrate stiffness does affect myogenic differentiation, implying that softer substrates can promote this process.^[^ ^50^ ^]^ Additionally, myosin/actin striations emerge later only on gels with stiffness typical of normal muscle (passive Young's modulus, *E* ≈12 kPa). While myotubes differentiate optimally on substrates with tissue‐like stiffness, it has been found that increased stiffness enhances migration and the proliferation of muscle stem cells.^[^ ^51^ ^]^ Our findings align with the fact that higher substrate stiffness corresponds to reduced myotube formation.

**Figure 7:**
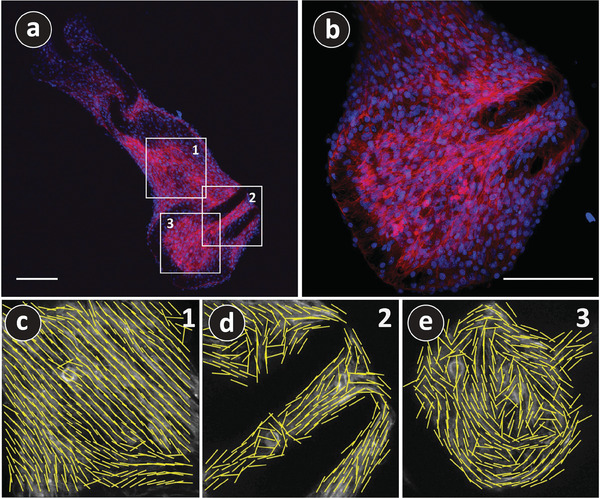
a) Low magnification and b) high magnification fluorescence images of C2C12 cells on architected PyC chain structure obtained at 900 °C. The nuclei were stained with Hoechst (blue), and actin fibers were visualized with phalloidin (red). The images were obtained with maximum intensity projection. c–e) The local orientation of actin filaments is represented by the yellow lines within an area of 5 µm^2^ of the cultured cells, obtained at the locations 1, 2, and 3 as indicated in (a), respectively. The scale bars in (a) and (b) are 200 µm. The results are derived from a single experiment.

Within 3D scaffolds, cells migrate and proliferate, extending inward to ultimately close or bridge the pores and create a sheet‐like tissue structure.^[^ ^52^, ^53^ ^]^ The collective behavior of cells, including the speed and final shape of the bridge while spreading in the 3D structure, depends on the geometry and size of the pores. For instance, the crowding or spreading of cells at the evolving interface is determined by whether the initial substrate is concave or convex.^[^ ^54^ ^]^ To explore the interaction between C2C12 cells and compliant PyC structures more extensively, the cells were cultured on PyC chain structures, which provide a variety of geometries for 3D cell growth. **Figure** **7**a,b shows low and high magnification maximum intensity projection fluorescence images of cultured C2C12 cells on a PyC chain structure. It can be seen here that the cells covered the entire chain structure, including the gaps between the lattice constructs and even within the revolute joints. Cells were observed to arrange themselves as flat sheets rather than aggregates, forming multiple cell layers within these sheets. Subsequently, they utilized intercellular connections to bridge the pores, spanning considerable distances and effectively filling the pore (see Figure 7a,b). Another important factor here is the alignment of the actin fibers of the cultured muscle cells. Actin rearrangement allows rapid cell shape change and cytoskeletal remodeling. Actin filaments generally align parallel to the direction of stress^[^ ^55^ ^]^ to adapt to the stress or reorient themselves according to the geometry of the substrate. On the other hand, myotubes, which consist of multiple bundles of muscle fibers, are mainly in parallel with actin filaments. Therefore, we investigated the actin fiber alignment of the C2C12 cells on the PyC chain structures using an algorithm introduced by Marcotti et al.^[^ ^56^ ^]^ The results, based on the maximum intensity projection of fluorescence images, are shown in Figure 7c–e and Movies S3–6. Following the method introduced in ref. [56], the order parameter spans from −1 to 1, with 1 indicating perfect alignment of actin filaments in a single preferred direction, −1 representing opposite alignment, and 0 denoting a random or isotropic distribution of orientations. We examined the alignment of actin in various layers of cells that formed bridges between the different parts of the chain. The space between the links (Region 1), the empty space within the revolute joint (Region 2), and the space contoured by the lattices (Region 3) were considered the regions of interest, as indicated in Figure 7a. The actin fibers exhibited a high degree of alignment, ≈90%, along the PyC chain length, as observed in Region 1 (Figure 7c). The higher degree of alignment could also be observed in the Z‐stack image analysis of Region 1, as shown in Movie S4, Supporting Information. The PyC chains featured parallel printing lines on their surfaces, which were also along the length of the chains, as shown in Figure S8, Supporting Information. These parallel printing lines might have guided the proliferation of the muscle cells along the chain length. Similar behavior was observed in Region 2 (Figure 7e), where aligned actin fibers were observed parallel to the printing line on the lattices of the revolute joints (see the lines in Figure S8b, Supporting Information). However, no clear trend of alignment was observed while analyzing the Z‐stack images of Region 2, as the order parameter was inconsistent (see Movie S5, Supporting Information). Interestingly, in Region 3, the actin fibers seemed to follow the closed contour of the PyC lattice link, as shown in Figure 7d. The cells, which were not in proximity to the contour, seemed to align randomly. Consequently, the Z‐stack analysis resulted in poor order parameters, as shown in Movie S6, Supporting Information. We are not sure about the reason behind it. One possible reason could be that only planar actin fibers could be tracked for Region 3, where the influence of the printing line should be minimal and the contour influence got maximized. However, detailed experiments are needed to understand the influence of 2D versus 3D geometry on the orientation of actin fibers.

## 3 Concluding Remarks and Future Research Directions

To summarize, microarchitected pyrolytic carbon structures were demonstrated for the first time for 3D cell growth of skeletal muscle cells. The PyC scaffolds were fabricated using micro‐stereolithography of microachitected resin structures, followed by pyrolysis. Apart from typical stationary and rigid 3D PyC scaffolds, we also fabricated novel compliant microarchitected PyC structures using a novel design‐based approach. The rigid 3D PyC structure was fabricated with a minimum design thickness of 15 µm, leading to a minimum PyC lattice thickness of 4.1 ± 0.1 µm, whereas the minimum design lattice thickness achieved for the microarchitected compliant 3D structures was 75 µm due to the need for support structures. Notably, the achieved geometrical dimensions of the PyC structures were comparable to those of skeletal muscle cells. The PyC material featured a highly amorphous microstructure with a small amount of graphitic content. The obtained PyC materials exhibited a temperature‐variant stiffness, with the highest stiffness of 29.57 ± 0.78 GPa for 900 °C. During cell culturing, skeletal muscle cells C2C12 exhibited a strong affinity to the PyC materials, showing good biocompatibility and cell proliferation. The cells populated within the rigid and compliant PyC scaffolds, leading to true 3D cell colonization. The 3D PyC structures further resulted in good alignment of the actin fibers of the cultured muscle cells along the compliant structure. A good number of myotubes were also formed on the PyC surface, which exhibited a dependency on the pyrolysis temperature‐dependent PyC stiffness. However, the myogenic differentiation capability of the PyC material was not fully realized here, which demands for further extensive investigation.

The findings of this study are significant for using 3D printed pyrolytic carbon structures as cell scaffolds. Particularly, the cell affinity to the compliant microarchitected PyC structures with innovative designs may open up new horizons for multifunctional scaffolds. Compliant PyC scaffolding structures could also allow for an in vitro culture strategy followed by minimally invasive surgical procedures, as the carbon chains and mechanisms could potentially be delivered through catheters or employing endoscopic procedures. Additionally, the multiscale fabrication strategy and musculoskeletal tissue engineering can lead to unique single‐step bone‐tendon‐muscle generation in the future.

## 4 Experimental Section

### Fabrication of 3D Printed Architected Pyrolytic Carbon Structures

Computer‐aided designs of a collection of microchains, micromechanisms, and interconnected lattice structures, representing the concepts of micromachines and designs for promoting shape‐morphing properties, were achieved using CAD software, including SolidWorks (developed by Dassault Systèmes) and Autodesk Inventor 2021. The CAD file of the Eiffel Tower was obtained from Thingiverse, an open‐access database for CAD files (https://www.thingiverse.com/thing:2795935). Once the designs were ready, conversion to.stl (standard tessellation language or stereolithography) file format allowed further slicing and processing. The master structures or “green parts” were additively manufactured using the Boston Micro‐Fabrication (BMF) MicroArch S130 laser micro‐stereolithography machine. A commercial epoxy resin from BMF (HTL yellow resin) was used as the photopolymerizable prototyping material. After slicing with the support of Chitubox software, an open‐access slicing software (https://www.chitubox.com/), the laser beam trajectories that polymerized the resin were defined. The laser worked in an additive, layer‐by‐layer process, activating a polymerization reaction and generating complex 3D structures. The printed structures were developed and washed in isopropanol for 15 min, and finally cured in a UV chamber for 1 h to achieve the desired properties.

The 3D printed epoxy structures were carbonized in a horizontal tube furnace (Carbolite Giro, Germany). The heat treatment protocol was adapted from the traditional heat treatment protocol in C‐MEMS.^[^ ^22^, ^57^, ^58^, ^59^, ^60^, ^61^ ^]^ Briefly, the heating protocol consisted of five stages: i) heating from room temperature to 350 °C with a heating ramp of 3 °C min^−1^; ii) isothermal heating at 350 °C for 3 h; iii) ramping from 350 °C to the final temperature with the ramp rate of 3 °C min^−1^; iv) isothermal heating at the final temperature for 2 h; and v) cooling down from the final temperature to room temperature using natural cooling. The final temperature was varied from 500 to 900 °C. A constant nitrogen gas flow (flow rate 80 L h^−1^) was maintained throughout the entire carbonization process.

### Characterization

The carbonized structures were imaged for inspection of any structural damage and deformities using an optical microscope (VHX‐100K Series Digital Microscope, Keyence, Germany). The lattice thickness of the printed structures was measured using Keyence software before and after carbonization. The shrinkage of the PyC structures was calculated using Equation (1), where *d*_Resin_ is the thickness of the fabricated resin lattice elements and *d*_Carbon_ is the lattice thickness of the corresponding structure after carbonization.

(1) $$\text{Shrinkage}\;\left(\%\right)=\frac{d_{\mathrm{Resin}}-d_{\mathrm{Carbon}}}{d_{\mathrm{Resin}}}\times 100$$

They were further investigated using scanning electron microscopy (SEM, Carl Zeiss AG‐SUPRA 60VP SEM) for detailed morphological characterization. The thermal degradation of the printed resin was investigated using thermogravimetric analysis, which was performed on a Mettler Toledo TGA2 Star system using a 5 °C min^−1^ heating rate under a nitrogen gas flow condition, and the data was acquired using STARe Excellence software. Raman spectroscopy was carried out on a Bruker Sentraa setup using a diode pumped solid state laser (λ = 532 nm) at 2 mW power with a penetration depth <1 µm. The Raman spectrum of architected PyC was deconvoluted using Voigt peak fitting using OriginPro software (OriginLab Corporation, USA), which also calculated the area under the curve for each peak. The crystallite size (L_a_) of the PyC material was calculated using the Tuinstra and Koenig equation, as proposed by Cançado et al.,^[^ ^62^ ^]^ which correlated L_a_ with the laser wavelength (λ) and integrated area ratio (A_D_/A_G_) of D‐band and G‐band, as shown in Equation (2).

(2) $$L_{a}=\left(2.4\times 10^{-10}\right)\lambda^{4}{\left(\frac{A_{D}}{A_{G}}\right)}^{-1}$$

### TEM Sample Preparation and Characterization

For TEM characterization of the architected PyC material, a cross‐sectional lamella was prepared by a focused ion beam (FIB, FEI Strata 400S) milling of an architected PyC with cubic unit cells with a lattice thickness of ≈60 µm (Figure S2, Supporting Information). The structure was fixed to an SEM stub by silver paste, and a protective layer of electron‐ and ion‐beam (EBID and IBID) induced platinum was deposited over the area of interest (20 ×  5 ×  3 µm^3^). Two trenches were milled by focused gallium ions, and the lamella was extracted using an Omniprobe micromanipulator. The lamella was then welded to the post of an Omniprobe copper grid and thinned to electron transparency (<100 nm) by the focused ion beam.

TEM characterization and EELS were carried out using an image‐side corrected FEI Titan G2 with a Gatan Tridiem 865 HR spectrometer. The microscope was operated at 300 kV, and the aberration coefficient (C_S_) was tuned to 5 µm. EELS data was acquired in TEM mode with a 2.5 mm (6.0 mrad) entrance aperture and an energy dispersion of 0.05 eV ch^−1^. For data treatment, Gatan Digital Micrograph 1.7 and origin were employed.

### In Situ Micromechanical Testing of PyC

The stiffness of architected PyC materials was characterized by compression tests on architected PyC micropillars. The micropillars featured a design diameter of 100 µm and height of 300 µm and were carbonized at different pyrolysis temperatures. Room temperature micropillar compression tests were carried out using a Hysitron triboindenter TI950 equipped with a 70 µm‐diameter flat punch diamond indenter. Micropillars were compressed under displacement control at displacement rates of ≈100 nm s^−1^ (corresponding to a constant strain rate of ≈10^−3^ s^−1^). Pillars were compressed up to a maximum strain of ≈15%, up to fracture, or up to the maximum load allowed by the load transducer (1.4 N), depending on the pillar's deformation behavior. The indenter displacement during pillar compression, which includes the deflection of the substrate, was corrected using Sneddon's correction (which considers that the pillar acts as a perfectly rigid flat‐punch indenter pushing into an isotropic half‐space^[^ ^63^ ^]^). Engineering stress (σ_eng_) and engineering strain (ε_eng_) were obtained using the pillar top diameter and length, respectively, which were measured in a FIB‐SEM microscope (Helios NanoLab 600i). Some pillars were compressed in situ inside the SEM using a Hysitron PI88 picoindeter equipped with a 90 µm‐diameter flat punch diamond indenter in order to record videos of the pillars' deformation during compression (videos of pillar compression are provided in Supporting Information).

### Skeletal Muscle Cell (C2C12) Culture

Skeletal muscle cells (C2C12) were cultured in a low‐endotoxin Dulbecco's modification of Eagle medium with 4.5 g L^−1^ glucose (PAN‐biotech, Germany) under growth conditions of 37 °C, 5% CO_2_ at about 90% humidity. The medium was supplemented with 10% fetal bovine serum (PAN‐biotech, Germany) and 1% penicillin/streptomycin (Sigma‐Aldrich, Germany).

To assess the cell culturing behavior with a PyC scaffolding structure, 3D printed lattice architectures based on cubic unit cells with truss thicknesses of 150 µm were used. The 3D PyC structures were prepared for cell culture by immersion in 70% ethanol for 1 h at room temperature. The scaffolds were thoroughly washed in 1× Dulbecco's Balanced Salt Solution (DPBS) (Sigma‐Aldrich, Germany) before incubation in a cell culture medium for 2 h at 37 °C and 5% CO_2_. For cell seeding, the scaffolds were placed in 35 mm glass bottom dishes (ibidi, Germany). Cells were seeded at a concentration of 10^5^ cells per dish. The cells on the 3D PyC structures were incubated in growth conditions for 7 days. The media was changed every 2–3 days. After 7 days, the media was changed to differentiation media consisting of very low endotoxin Dulbecco's modification of Eagle medium with 4.5 g L^−1^ glucose supplemented with 10% horse serum (Gibco life technologies, USA) and 1% penicillin/streptomycin. The media was changed every 2–3 days. After 7 or 22 days respectively, the cells were fixed using 4% paraformaldehyde (Sigma, Germany) for 1 h at room temperature. In addition, PyC chain structures with a design lattice thickness of 150 µm were tested for cell culturing experiments to demonstrate the feasibility of using compliant architected PyC as innovative tissue engineering scaffolds.

The biocompatibility of the structures was assessed following the ISO 10993 standard, MTT assay. In summary, the structures were incubated in 1 mL of culture medium (DMEM supplemented with 10% FBS and 1% penicillin/streptomycin) at 37 °C for 72 h to create extractions. Next, 10,000 Rat Embryonic Fibroblast wild‐type (REF52wt) cells were cultured with 100 µL of medium for 24 h. Afterward, the medium was replaced with 100 µL of extraction medium, and the cells were further incubated at 37 °C for 24 h. To evaluate the number of viable cells, 50 µL of methylthiazolyldiphenyl‐tetrazolium bromide (MTT; Sigma‐Aldrich, Germany) solution was added, and the absorbance was measured at 570 and 620 nm for reference after a 4‐h incubation. Negative control samples were prepared using fresh and untreated culture medium, while positive control samples contained medium with 20% dimethyl sulfoxide. The results were normalized to the absorbance measured in the control samples. This entire procedure was repeated five times for technical replicates, and three independent experiments were conducted.

### Fluorescence Staining and Imaging of Cultured Cells

For fluorescent staining, the cells were permeabilized by incubation with 0.2% Triton‐X100 (Sigma, Germany) in DPBS for 10 min at room temperature, followed by thoroughly washing with DPBS. To prevent unspecific binding, the samples were incubated for 1 h in a blocking buffer consisting of 3% w/v bovine serum albumin (Sigma‐Aldrich, Germany), 0.1% Tween‐20 (Sigma‐Aldrich, Germany) and 10% fetal bovine serum. For labeling of the myosin heavy chain, the samples were incubated overnight at 4 °C with anti‐myosin heavy chain antibody (#A44558, antibodies, UK) at a concentration of 1:200 in blocking buffer. The samples were washed with 0.1% Tween‐20 in DPBS, followed by incubation with goat Anti‐Mouse IgG H&L (Alexa Fluor 488) (#ab150113, Abcam, UK) at a concentration of 1:1000 in blocking buffer for 1 h at room temperature. For actin labeling, the samples were incubated with Alexa Fluor 647 phalloidin (Invitrogen, USA) at a concentration of 1:1000 in a blocking buffer for 1 h at room temperature. After washing with 0.1% Tween‐20 in DPBS, the samples were incubated with 10 µg mL^−1^ Hoechst 33258 (Invitrogen, USA) in blocking buffer for 20 min at room temperature, followed by washing. Confocal imaging was performed using a Nikon AX confocal microscope located at the Nikon Imaging Center Heidelberg. Actin alignment characterization has been performed by utilizing a fast Fourier transform (FFT) of square windows of 18 × 18 pixels chosen from the image in the original space. The windows are overlapped by 50%, and an intensity threshold of 68 was considered to filter the blank spaces. The analysis was based on the algorithm presented in Marcotti's article.^[^ ^56^ ^]^ The used python script was adapted from https://github.com/OakesLab/AFT‐Alignment_by_Fourier_Transform/tree/master/Python_implementation. To investigate the muscle cells and myotubes on the 3D and 4D PyC scaffolds, the cells were fixed with paraformaldehyde (Sigma, Germany) and dried with a critical point dryer (Leica Em CPD300, Germany). Thin titanium and gold layers (5 and 10 nm, respectively) were sputtered on the samples prior to SEM (Jeol, 5 kV).

## Conflict of Interest

The authors declare no conflict of interest.

## Supporting information

Supporting Information

Supplemental Movie 1

Supplemental Movie 2

Supplemental Movie 3

Supplemental Movie 4

Supplemental Movie 5

Supplemental Movie 6

## Data Availability

The data that support the findings of this study are available on request from the corresponding author. The data are not publicly available due to privacy or ethical restrictions.
