# Supplementary material for: Microarchitected Compliant Scaffolds of Pyrolytic Carbon for 3D Muscle Cell Growth
Source: Adv Healthc Mater. 2024 Jan 2;13(9):2303485. doi: 10.1002/adhm.202303485 (PMC11469158; doi:10.1002/adhm.202303485)
Supplement: Supplementary file 1 — Supporting Information [file ADHM-13-2303485-s002.pdf]

ADVANCED HEALTHCARE MATERIALS

Supporting Information

for *Adv. Healthcare Mater.*, DOI 10.1002/adhm.202303485

Microarchitected Compliant Scaffolds of Pyrolytic Carbon for 3D Muscle Cell Growth

Mohammadreza Taale, Barbara Schamberger, Miguel A. Monclus, Christian Dolle, Fereydoon Taheri, Dario Mager, Yolita M. Eggeler, Jan G. Korvink, Jon M. Molina-Aldareguia, Christine Selhuber-Unkel, Andrés Díaz Lantada and Monsur Islam**

Supporting information: Microarchitected compliant carbon scaffolds for 3D muscle cell growth

Mohammadreza Taale Barbara Schamberger Miguel Monclus Christian Dolle Fereydoon Taheri Dario Mager Yolita M. Eggeler Jan G. Korvink Jon M. Molina-Aldareguia Christine Selhuber-Unkel Andrés Díaz Lantada Monsur Islam**

Dr. Mohammadreza Taale, Dr. Barbara Schamberger, Dr. Fereydoon Taheri, Prof. Dr. Christine Selhuber-Unkel
Institute for Molecular Systems Engineering and Advanced Materials (IMSEAM), Heidelberg University, Im Neuenheimer Feld 225, 69120 Heidelberg, Germany.

Dr. Miguel A. Monclus
IMDEA Materials Institute, Eric Kandel, 2, Getafe, 28906, Spain.

Dr. Christian Dolle, Prof. Dr. Yolita Eggeler
Microscopy of Nanoscale Structures & Mechanisms (MNM), Laboratory for Electron Microscopy (LEM), Karlsruhe Institute of Technology, Engesserstr. 7, D-76131, Karlsruhe, Germany.

Dr. Dario Mager, Prof. Dr. Jan G. Korvink
Institute of Microstructure Technology, Karlsruhe Institute of Technology, Hermann-von-Helmholtz-Platz 1, 76344 Eggenstein-Leopoldshafen, Germany.

Prof. Dr. Jon M. Molina-Aldareguia
Department of Mechanical Engineering, Universidad Politécnica de Madrid, José Gutierréz Abascal, 2, Madrid, 28006, Spain.
IMDEA Materials Institute, Eric Kandel, 2, Getafe, 28906, Spain.

Prof. Dr. Andrés Díaz Lantada
Department of Mechanical Engineering, Universidad Politécnica de Madrid, José Gutiérrez Abascal 2, 28006 Madrid, Spain.
Email Address: andres.diaz@upm.es

Dr. Monsur Islam
Institute of Microstructure Technology, Karlsruhe Institute of Technology, Hermann-von-Helmholtz-Platz 1, 76344 Eggenstein-Leopoldshafen, Germany.
IMDEA Materials Institute, Eric Kandel, 2, Getafe, 28906, Spain.
Email Address: monsurislam79@gmail.com (M.I.)

Supporting information

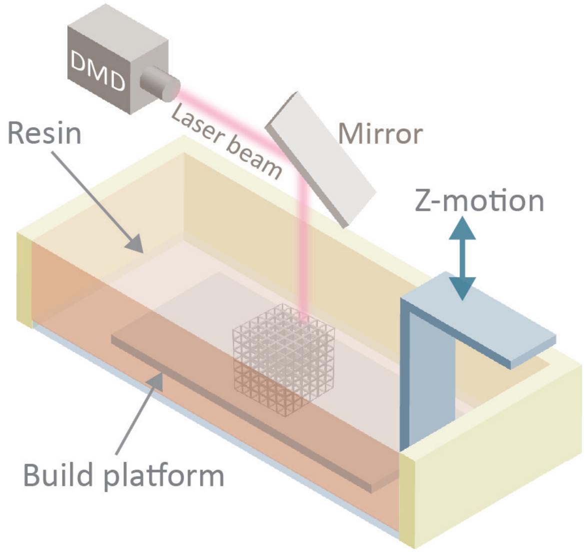

Figure SI1: Schematic of the top-down micro-stereolithography process used in our work.

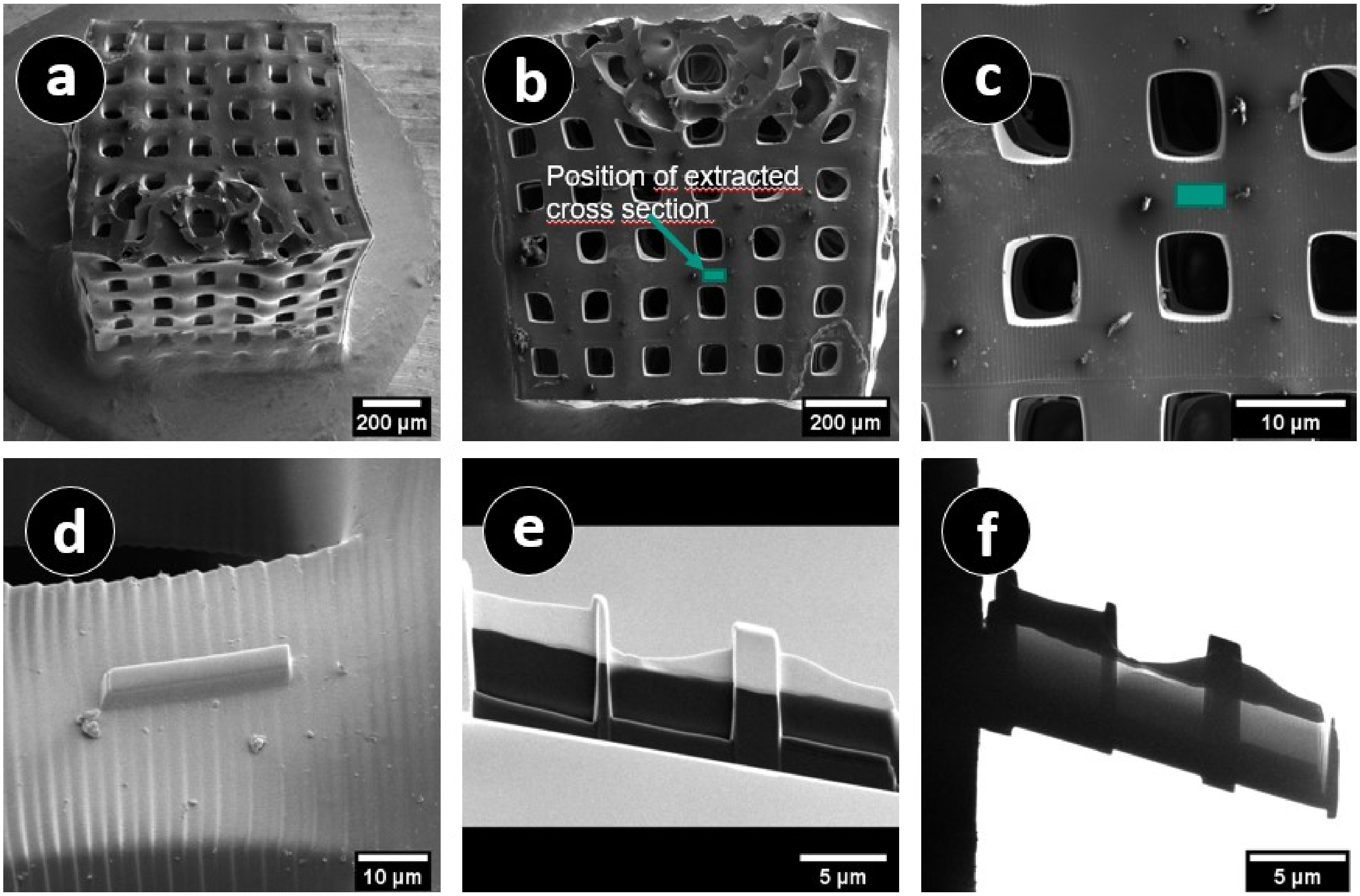

Figure SI2: Sample preparation for TEM characterization of architected PyC material. (a) Architected pyrolytic carbon with cubic unit cells with a PyC lattice thickness of ∼60 µm fixed on an SEM stub using silver paster for focused ion beam (FIB) milling. The architected PyC was achieved for the pyrolysis temperature 900°C. (b) Low and (c) high magnification of the location of the architected PyC from where FIB was performed to extract the thin lamellas. (d) The PyC lattice after FIB milling, showing the milled location. (e) SEM and (f) TEM image of the FIB-milled lamella of the PyC material fixed to a copper grid.

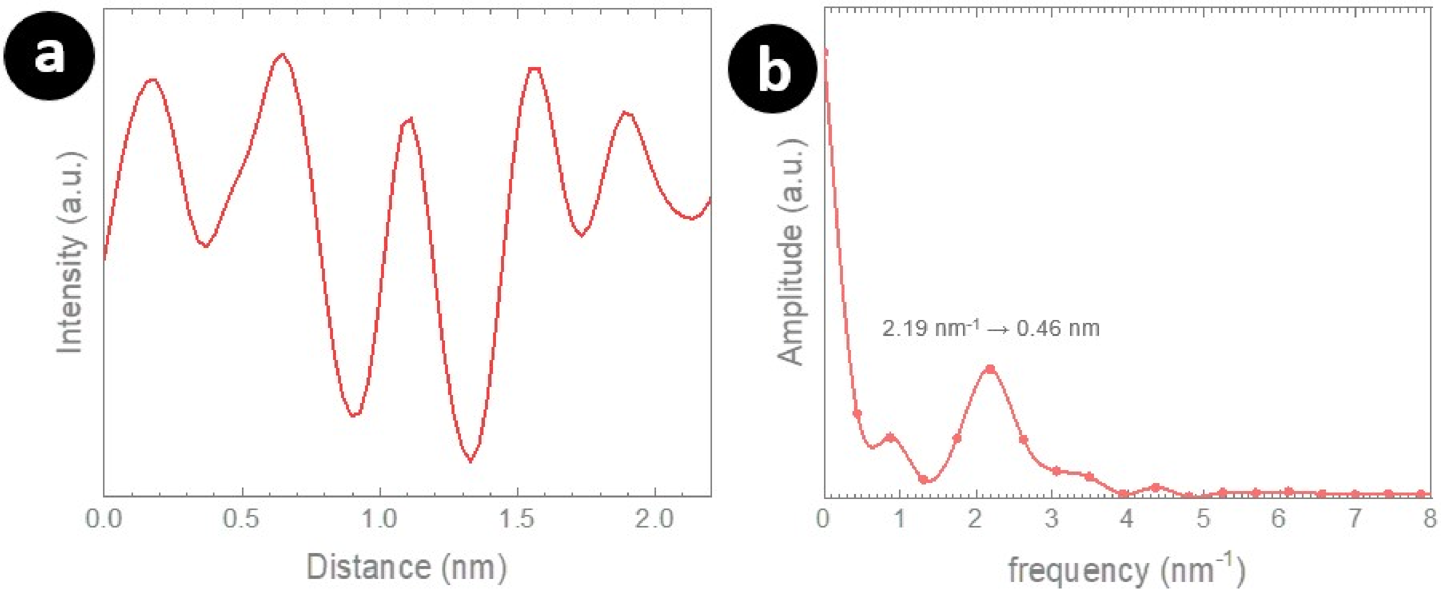

Figure SI3: (a) Intensity measurement of the line-scan and (b) FFT of the line-scan of the graphitic fringes shown in Figure 2c, measuring the interlayer spacing of the graphitic fragments.

Temperature	Diameter (µm)	Height (µm)	Aspect ratio
500°C	41.7±1.2	119.0±1.7	2.9±0.1
700°C	33.5±0.7	104.5±3.5	3.1±0.2
900°C	31.0±3.5	102.3±3.8	3.3±0.3

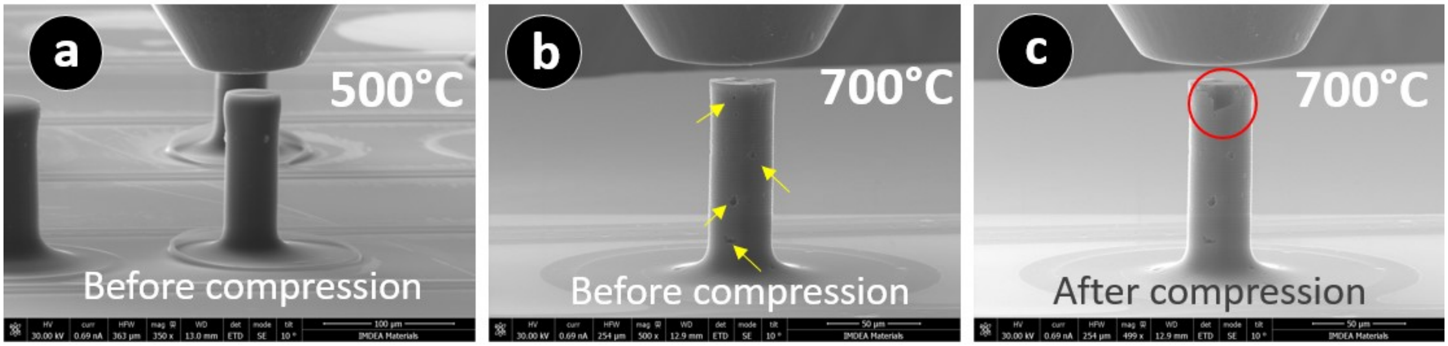

Figure SI4: The top table presents the dimensions and aspect ratio of the PyC micropillars used for micromechanical testing. SEM images of the PyC micropillars obtained at the pyrolysis temperature (a) 500°C and (b) 700°C (middle) before the compression tests. The arrows in (b) indicate surface defects in the micropillars for the pyrolysis temperature 700°C. (c) The micropillar for 700°C after the compression test, where the red circle indicates the mechanical failure site.

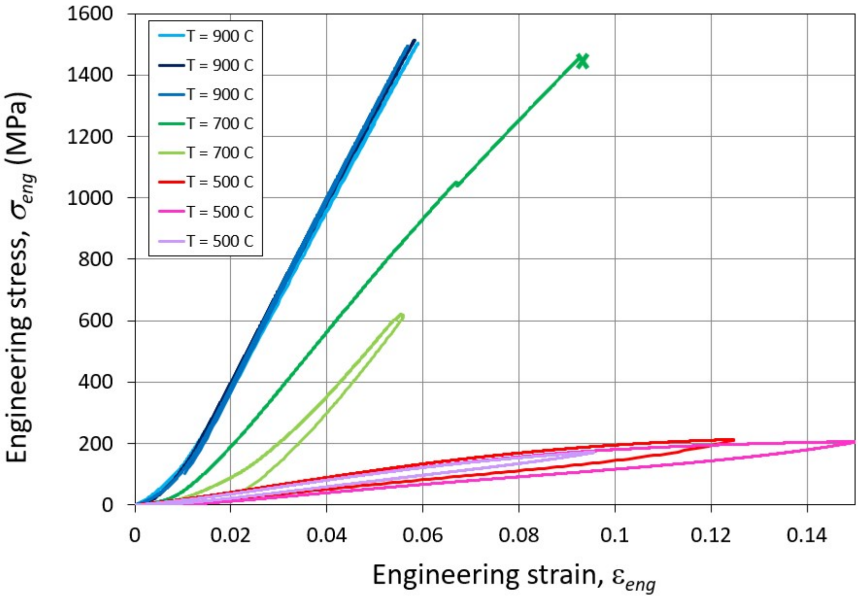

Figure SI5: Engineering stress versus engineering strain curves of multiple PyC micropillars used at different temperatures, showing the reproducibility of the tests.

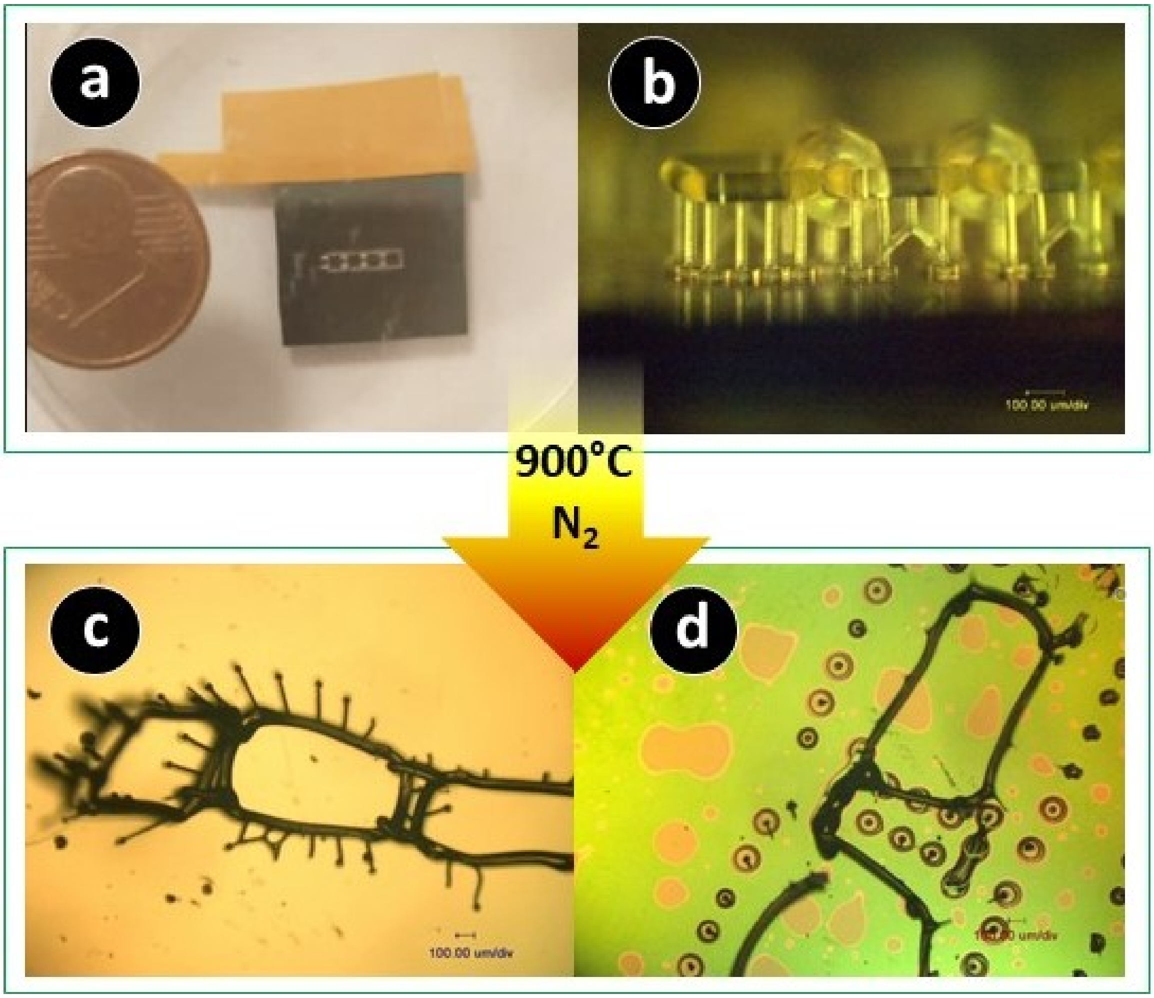

Figure SI6: (a) An example of printed chain structure. (b) The support structures, which facilitated the printing of the chain structures. (c) and (d) the failed carbonized chain structures that occurred due to the asymmetric forces exerted by the supports.

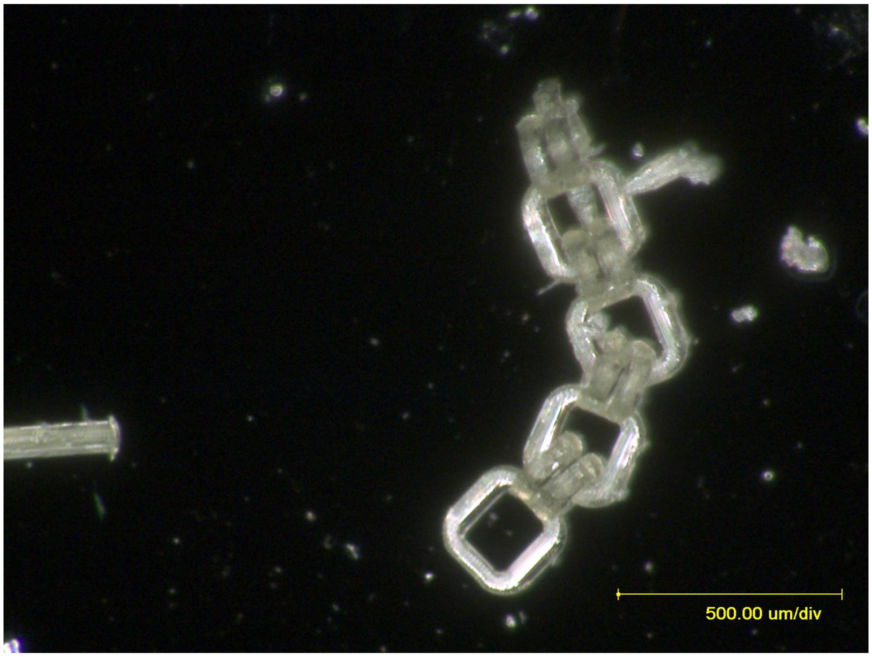

Figure SI7: 3D printed resin chain structure with the lattice thickness of 50 µm.

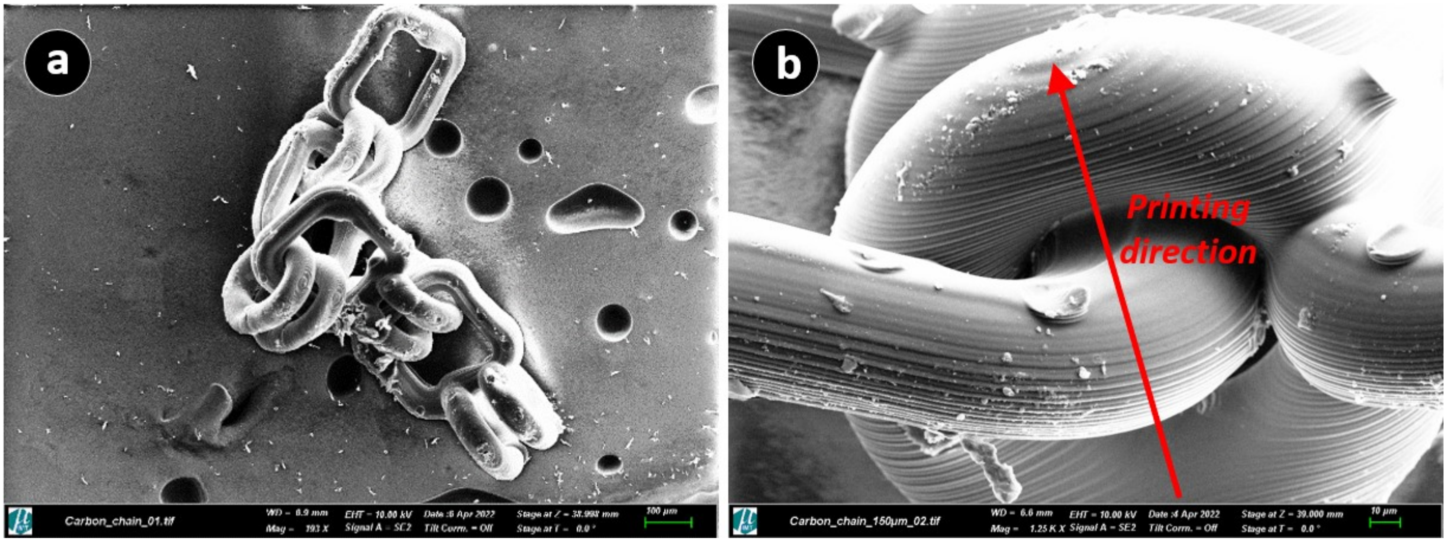

Figure SI8: (a) SEM of a 3D PyC chain structure, obtained at the pyrolysis temperature of 900°C. (b) High magnification SEM image of the PyC chain structure, showing the printing lines on its surface.

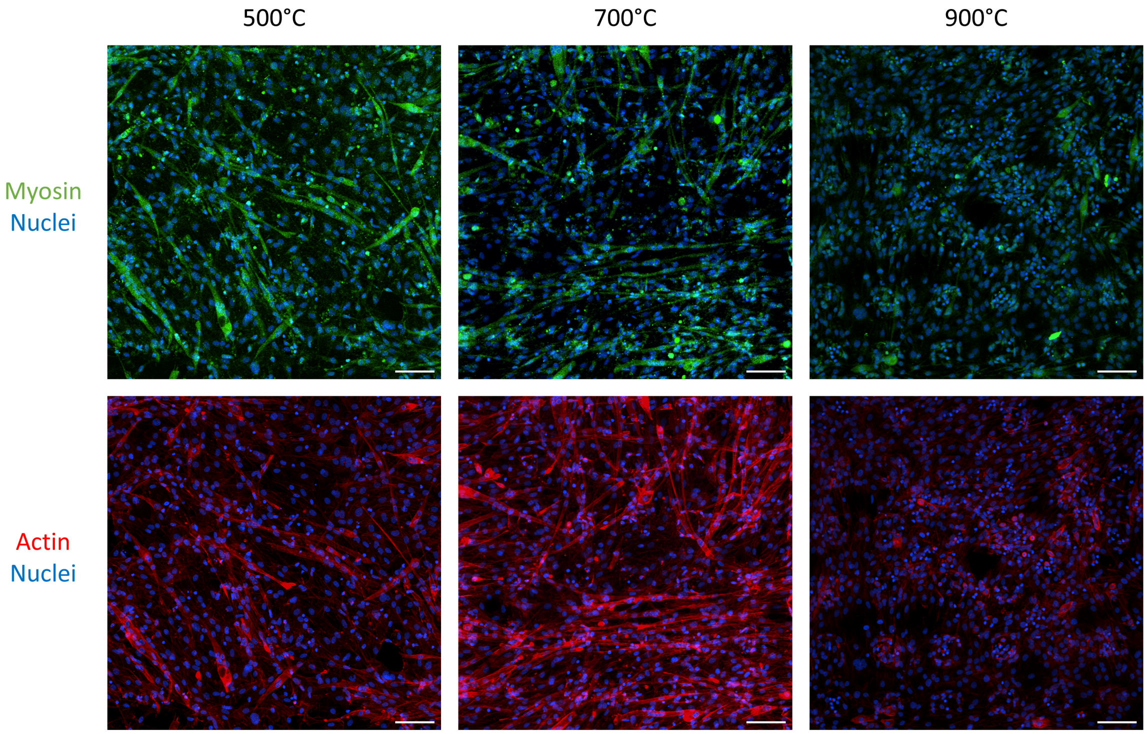

Figure SI9: Maximum intensity projections of fluorescence images of C2C12 cells on architected 3D PyC structures pyrolyzed at 500°C, 700°C, 900°C. The nucleus was stained with Hoechst (blue), actin fibers were stained with phalloidin (red), and the myotubes were visualized with anti-myosin heavy chain antibody and Alexa Fluor 488 (green). The scale bars are 100 µm.

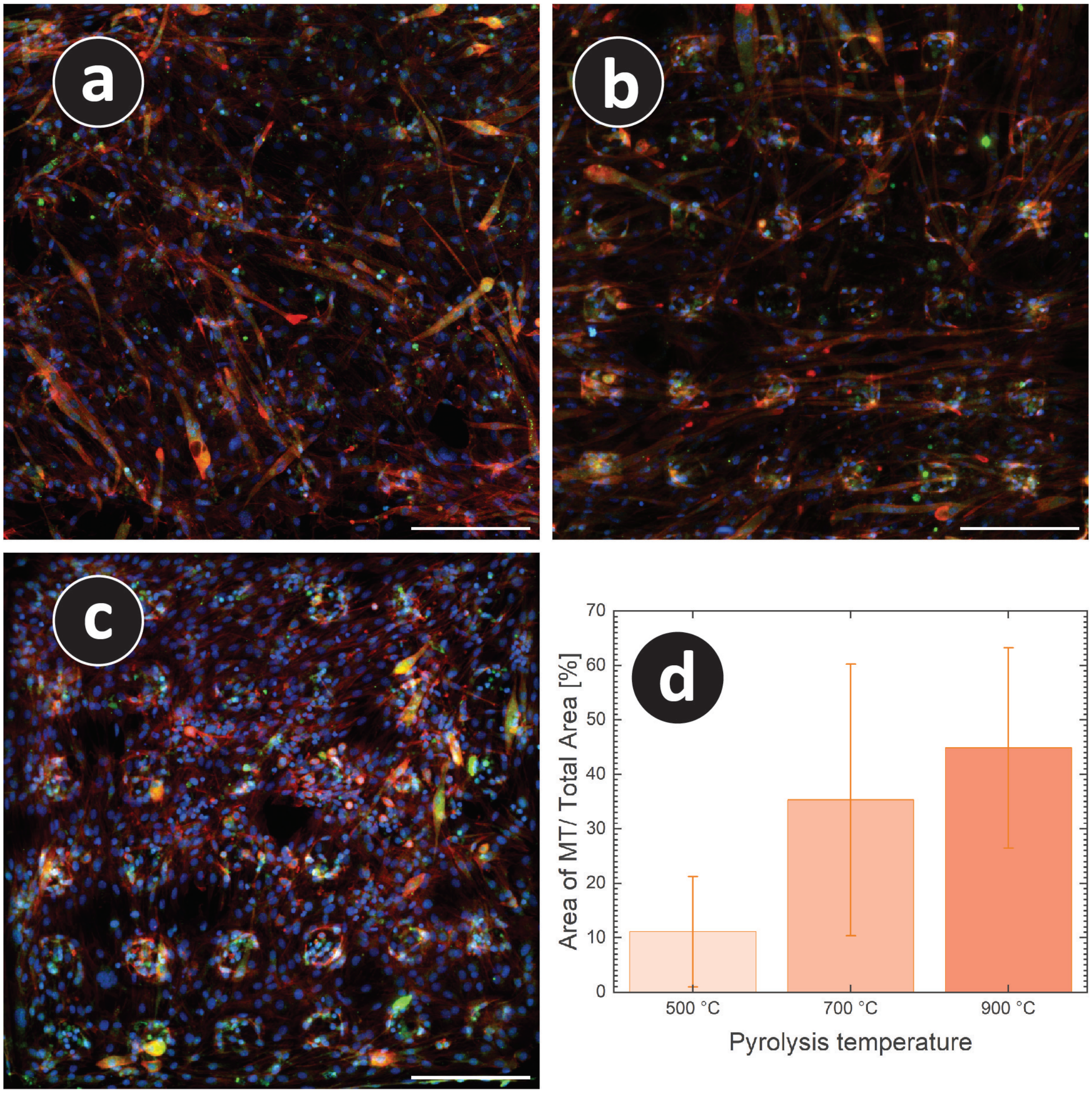

Figure SI10: Maximum intensity projections of fluorescence images of C2C12 cells on architected 3D PyC structures pyrolyzed at (a) 500°C, (b) 700°C, (c) 900°C. The nucleus was stained with Hoechst (blue), actin fibers were stained with phalloidin (red), and the myotubes were visualized with anti-myosin heavy chain antibody and Alexa Fluor 488 (green). (d) Illustrates the area ratio of the myotube formed on one side of the material. The scale bars in (a-c) are 200 µm.
